# Summary of best evidence for safe management of vasopressors through peripheral intravenous catheters

**DOI:** 10.1186/s12912-025-03635-3

**Published:** 2025-07-31

**Authors:** Guanjie Chen, Chen Shen, Chenwei Pan, Xiaohui Gao, Mingzhu Sun, Xiaoqing Li

**Affiliations:** 1 Jiangsu Provincial Key Laboratory of Critical Care Medicine, Department of Critical Care Medicine, Zhongda Hospital, Southeast University, 87 Dingjiaqiao, Gulou District, Nanjing, 210009 P.R. China; 2 Department of Critical Care Medicine, Lianyungang First People’s Hospital, Lianyungang, China

**Keywords:** Vasopressors, Peripheral intravenous catheters, Intravenous administration, Complications, Evidence-based nursing

## Abstract

**Background:**

Vasopressors are critical for maintaining hemodynamic stability in critically ill patients, traditionally administered via central venous catheters (CVCs). However, CVCs carry risks of complications and insertion delays. Peripheral intravenous catheters (PIVCs) offer a rapid alternative but pose risks of extravasation and phlebitis. This study aimed to evaluate and summarize the evidence for the safe management of vasopressors through peripheral intravenous catheters, providing reference for clinical practice.

**Methods:**

This evidence summary utilized the standard evidence summary report of Fudan University Center for Evidence-based Nursing, which includes problem establishment, evidence retrieval, literature screening, quality evaluation of the literature, the summary and grading of evidence. The registration number is “ES20246694”. Current literatures were systematically searched for the best evidence for safe management of vasopressors through PIVCs. BMJ Best Practice, UpToDate, DynaMed, Joanna Briggs Institute, Cochrane Library, Guidelines International Network, National Institute for Health and Clinical Excellence, Scottish Intercollegiate Guidelines Network, Registered Nurses’ Association of Ontario, Intravenous Nurses Society, Chinese Nursing Association, PubMed, Embase, CINHAL, Web of Science, Chinese Medical Journal Full-text Database, Sinomed, CNKI, Wanfang, and VIP were searched from database establishment to 27 June 2025. Literature types included clinical practice guidelines, clinical decisions, expert consensuses, systematic reviews, and evidence summaries.

**Results:**

Our systematic search retrieved 1,925 publications, and finally identified 12 articles that had high-quality results. The evidence synthesis comprised three clinical decisions, four guidelines, one expert consensus, and four systematic reviews. We summarized the 29 pieces of best evidence from these articles, covering five aspects: training and education, infusion site selection, vascular access placement, infusion regimen optimization, and complication management. Of these pieces of evidence, 23 were ‘strong’ and 6 were ‘weak’, 9 pieces of evidence were recommended in level one.

**Conclusion:**

The following 29 pieces of evidence for safe management of vasopressors through peripheral intravenous catheters were finally recommended. However, due to the multinational origin of the evidence, feasibility, appropriateness, clinical significance, and effectiveness must be evaluated within institutional contexts prior to implementation.

**Supplementary Information:**

The online version contains supplementary material available at 10.1186/s12912-025-03635-3.

## Introduction

Vasopressors are medications that constrict blood vessels by activating receptors on vascular smooth muscle, maintaining blood pressure and organ perfusion. They are foundational drugs in managing patients with circulatory failure, such as shock [1]. Traditionally, the central venous catheter (CVC) is considered the gold-standard route for vasopressor infusion due to its large lumen and high blood flow [2]. However, CVC placement is complex, requiring specialized skills and specific settings. The failure rate is 2.04%. The procedure can cause serious complications, including arterial cannulation (0.28%), arterial puncture (1.62%), and pneumothorax (0.44%) [3]. Furthermore, CVC insertion often causes significant treatment delays in emergencies [4]. Each hour of delay in starting vasopressors is associated with an approximately 5.3% increase in patient mortality risk [5]. CVCs also carry risks like catheter-related bloodstream infection and deep vein thrombosis. An estimated 3% of patients develop at least one serious complication within 3 days of CVC placement [3].

Recent evidence suggests peripheral intravenous catheters (PIVCs) can safely deliver vasopressors when proper protocols are followed [6]. The PIVC is a catheter inserted and secured within a peripheral vein. This route is particularly suitable during the initial resuscitation phase for hemodynamically unstable patients. PIVC placement should be prioritized over delaying treatment while awaiting CVC insertion [4, 7].PIVC insertion is relatively simple and rapid, significantly reducing medication initiation time. The median time reduction is nearly 100 min [8]. This allows patients to achieve target blood pressure earlier, reduces mortality, and shortens mechanical ventilation duration [9]. Additionally, PIVC use eliminated the need for a CVC in 37% of patients, reducing risks and costs associated with that more invasive procedure. It avoided direct supply costs of $8,900 [10] and expanded the settings where vasopressors can be safely initiated, including the emergency department, general wards, and even resource-limited environments [11].

However, administering vasopressors through PIVCs is not without risks. The primary concern is extravasation. Extravasation is the accidental leakage of irritant or vesicant solutions into the surrounding tissues outside the vascular lumen [7]. A meta-analysis [12] showed an overall complication rate of approximately 7% for vasopressor infusion through PIVCs, with extravasation events accounting for 72% of these. Although the vast majority of extravasations are mild in nature, and severe tissue damage like necrosis is very rare [13], extravasation can still cause local tissue ischemia, necrosis, and functional impairment [14]. A core challenge in current clinical practice is the lack of a unified, specific standardized protocol for vasopressor infusion through PIVC. The survey [15] reveals significant variations in nursing practices regarding site rotation, vessel selection, flow rate adjustment, infusion pump use, and catheter maintenance. Existing guidelines are often too broad. They lack clear, actionable details on critical aspects such as maximum safe drug concentrations, criteria for optimal catheter size selection, specific infusion technique parameters, and systematic complication management protocols. This absence of standardized protocols is a key barrier limiting the safe and widespread use of PIVCs for vasopressor infusion. It represents a significant gap in current knowledge and practice.

As evidence supporting the safety of PIVC use accumulates and medical devices advance, there is an urgent need to systematically summarize and evaluate the quality of existing research. This will bridge the evidence-practice gap. This study aims to use an evidence summary approach. It will comprehensively search for, critically appraise, and integrate the best available evidence on vasopressor administration through PIVC. It will focus on key aspects of standardized procedures, including catheter selection, drug preparation, infusion monitoring, and complication prevention and management. Evidence summaries can condense dispersed findings from various studies into a standardized framework, and transform fragmented knowledge into clear, actionable guidelines. The findings will provide a direct basis for developing or refining specific, actionable clinical practice standards for vasopressor infusion through PIVCs. This will reduce patient complications and mortality, decrease CVC use and related costs, shorten medication delays, and guide nursing and medical education.

## Methods

This evidence summary utilized the standard of evidence summary report Fudan University Center for Evidence-based Nursing, which includes problem establishment, evidence retrieval, literature screening, quality evaluation of the literature, the summary and grading of evidence [16]. The registration number is ‘ES20246694’.

### Problem establishment

This study focused on the clinical question of ‘How to safely infuse vasopressors through PIVCs?‘. This question spans multiple steps and settings, requiring consideration of standardized procedures, staff roles, implementation locations, and evidence types. While the traditional PICO model (Population, Intervention, Comparison, Outcome) is widely used for evaluating treatment efficacy, it lacks definitions for key evidence translation elements. Therefore, this study employs the PIPOST model (Population, Intervention, Professional, Outcome, Setting, Type of evidence) [17], proposed by the Fudan University Center for Evidence-based Nursing, as shown in Table 1.

**Table 1:** Criteria used in the summary of evidence

PIPOST	Description
Population	Adult patients who received vasopressors through PIVC
Intervention	Training and education, infusion site selection, vascular access placement, infusion regimen optimization, and complication management
Professional	Medical staff
Outcome	Incidence of drug extravasation, phlebitis, and other complications
Setting	General wards, acute and critical care units, operating rooms, etc.
Type of evidence	Clinical practice guidelines, clinical decisions, expert consensuses, systematic reviews, and evidence summaries

### Evidence retrieval

According to the evidence pyramid ‘6S’ model, the search was performed from top to bottom. The following databases were searched: BMJ Best Practice, UpToDate, DynaMed, Joanna Briggs Institute(JBI), Cochrane Library, Guidelines International Network(GIN), National Institute for Health and Clinical Excellence (NICE), Scottish Intercollegiate Guidelines Network(SIGN), Registered Nurses’ Association of Ontario (RNAO), Intravenous Nurses Society(INS), Chinese Nursing Association, PubMed, Embase, CINHAL, Web of Science, Chinese Medical Journal Full-text Database, Sinomed, CNKI, Wanfang, and VIP.

References were traced in addition to the aforementioned databases. The search was performed using a combination of subject words and free words. The search words were“vasoconstrictor agents, vasoconstrictor*, vasopressor*, vasoactive agonist*, vasoactive agent*, epinephrine, norepinephrine, dopamine, dobutamine, peripheral catheterization*, peripheral venous catheter*, peripheral intravenous, and indwelling needle”. The search period was from database establishment to 27 June 2025. We used the search strategy described in the supplementary material 1.

### Inclusion and exclusion criteria of evidences

Inclusion criteria for this study were (1) study subjects were patients ≥ 18 years who received vasopressors through PIVC; (2)literatures focused on the safe management of infusion of vasopressors through PIVC, including training and education, selection of infusion site, placement of vascular access, reasonable infusion regimen and management of complications; (3)literature types were clinical practice guidelines, clinical decisions, expert consensus, systematic review, and evidence summary.

Exclusion criteria were(1)literature language was not Chinese and English, considering database coverage and search feasibility, constraints in language capabilities and resources, and the generalizability and academic impact of the evidence; (2) literature information was incomplete or unavailable; (3) literatures were directly translated or repeatedly included; (4) literatures with failed quality evaluation.

### Literature screening

Two researchers trained in evidence-based medicine independently screened the literatures using a predetermined, standardized manual for inclusion and exclusion criteria. The obtained literatures were imported into Noteexpress and duplicates were deleted. The titles, abstracts, and keywords were read for preliminary screening, the full texts were read for rescreening, and the quality of rescreened literatures were evaluated. Disagreements were resolved by re-examining the original source, discussing their reasoning, and consulting a third intravenous field specialist for final adjudication.

### Quality evaluation of the literature

Given the variations in content, structure, and methodology across different document types, appropriate critical appraisal tools were selected to assess the quality of each type of literature. Before evaluation, an evidence-based nursing expert interpreted the specific criteria for each tool, providing illustrative examples. For guidelines, four researchers independently performed appraisals. Inter-rater reliability was assessed using the intraclass correlation coefficient (ICC). ICC values range from 0 to 1, with values closer to 1 indicating higher agreement; an ICC > 0.75 was considered good agreement [18]. For systematic reviews, expert consensuses, clinical decisions, and evidence summaries, the quality appraisal was conducted independently by two researchers. Disagreements were resolved by re-examining the source document, discussing their rationale, and consulting a third intravenous field specialist for final adjudication.

Appraisal of Guidelines for Research and Evaluation (AGREE II) was used to evaluate the quality of the guidelines [19]. AGREE II evaluation system consists of 6 fields and 23 items. Each item is scored from 1 to 7, with higher scores indicating that the guideline is more consistent with that item. Standardized scores in each field = [(actual score evaluated − lowest possible score)/(highest possible score − lowest possible score)] × 100%. According to the score of each domain, the recommendation level is determined. All six fields scored ≥ 60% were Grade A (strong recommendation), ≥ 3 fields scored ≥ 30% were Grade B (moderate recommendation), and ≥ 3 fields scored < 30% were Grade C (non-recommendation). However, AGREE II assesses only the rigor of the guideline development process, not the validity or reliability of the recommendations themselves. Furthermore, its results are susceptible to the individual interpretation and experience of the appraisers.

The literature quality assessment for systematic reviews and expert consensus was conducted using the tool by the Australian JBI Centre for Evidence-Based Health Care [20]. Quality was assessed by judging each item as “Yes,” “No,” “Unclear,” or “Not Applicable.” However, the JBI tool exhibits considerable subjectivity in interpreting some items, and its qualitative nature limits the precise quantification of quality.

For evidence summaries and clinical decisions, the Critical Appraisal for Summaries of Evidence (CASE) tool [21] was applied to evaluate the quality. This tool contains four domains and ten items. Quality was assessed by judging each item as “Yes,” “Not completely,” or “No.” Similarly, the CASE tool is highly susceptible to appraiser subjectivity, and its qualitative approach makes precise quality quantification or comparative assessment difficult.

### Evidences extraction and summary

Literature translation was performed by two researchers with nursing backgrounds who had passed the medical English test system (METS) Level 4, ensuring accuracy and reliability. Using a structured extraction form (including evidence topic, description, source, reference, and level), both researchers independently completed evidence extraction and synthesis. Disagreements were resolved by re-examining the source documents with focused verification. Final unresolved disagreements were referred to a third intravenous field specialist for adjudication.

Evidence synthesis followed these principles: (1) For overlapping evidence, concise and clear recommendations were prioritized; (2) Complementary evidence was logically integrated; (3) Conflicting evidence was resolved by prioritizing newer publications and higher-quality evidence.

JBI evidence pre-classification system [22] classified evidence into levels 1–5 based on study design. Level 1 evidence comprises randomized controlled trial studies, Level 2 quasi-experimental studies, Level 3 observational studies, Level 4 descriptive studies, and Level 5 encompasses expert opinion and basic research. For clinical decisions and guidelines, original sources were traced for grading. Evidence from summaries directly retained their original classifications.

Using the JBI evidence rank system [22], an expert panel graded recommendations as A (strong) or B (conditional) based on assessments of feasibility, appropriateness, clinical meaningfulness, and effectiveness. Strong recommendations (Grade A) require evidence that unequivocally demonstrates benefits outweigh harms (or vice versa), derives from higher-quality studies, represents reasonable resource allocation, and has fully considered patient values, preferences, and experiences. Conditional recommendations (Grade B) apply when evidence is of lower quality, benefits versus harms are uncertain, resource implications are unclear, or patient values, preferences, and experiences lack comprehensive consideration. The panel comprised eight experts: two evidence-based methodology specialists and six clinical experts (one operating room chief physician, one associate chief nurse from critical care, and four intravenous therapy specialists). Educational backgrounds included two PhDs (25.0%), three master’s degrees (37.5%), and three bachelor’s degrees (37.5%). All experts had over 10 years of field experience, ensuring representative and reliable conclusions.

## Results

### Search results

**Fig. 1:**
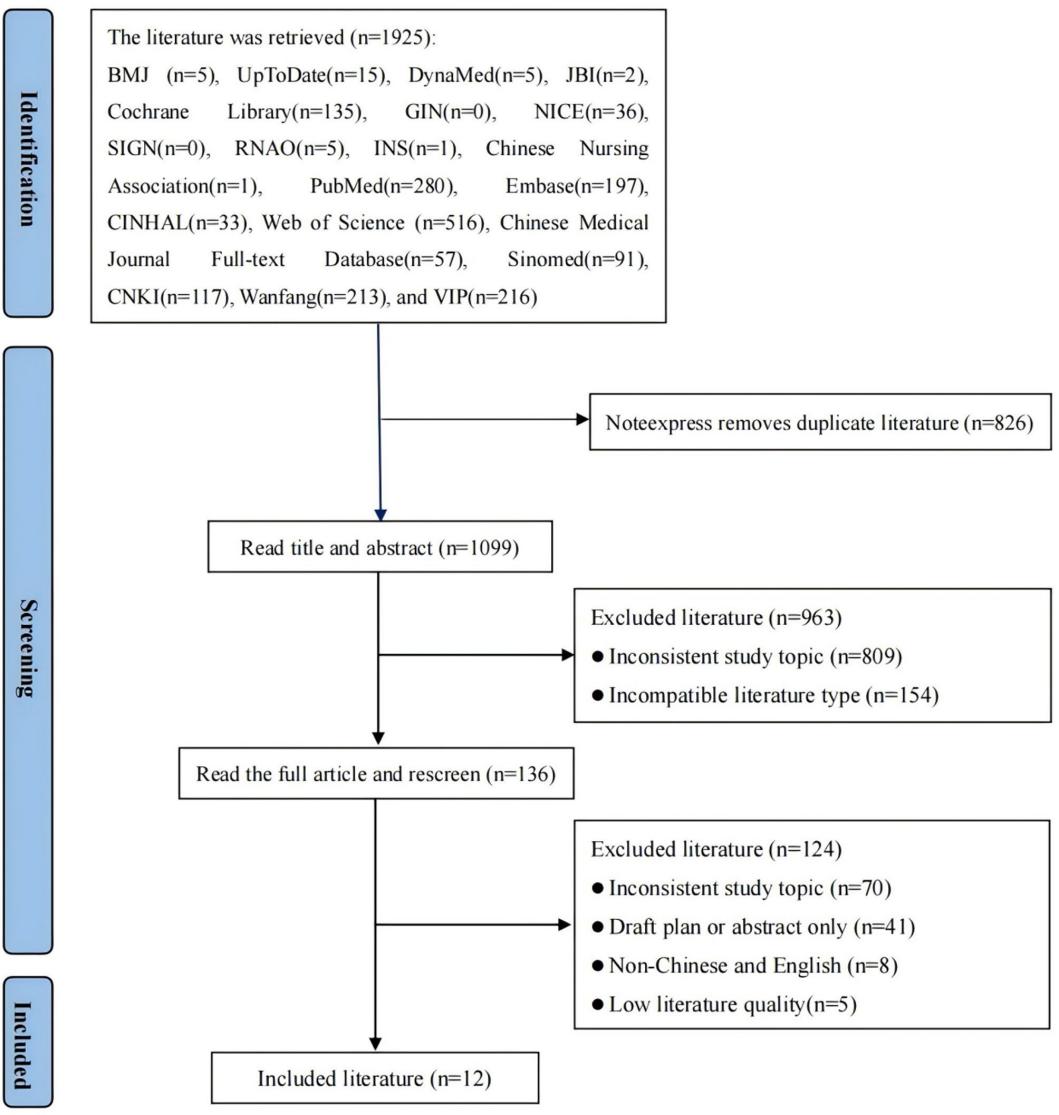
Flow chart of literature screening

A total of 1925 literature items were retrieved in this study. After import to Noteexpress and removal of duplicates, 1099 articles remained. Two team members independently read the title and abstract of each article. After excluding literature without full articles and unrelated items, 136 articles remained. Further reading of the full text and removal of 124 articles that did not meet the inclusion criteria of this study resulted in 12 articles included in the final analysis (Fig. 1). These 12 articles comprised three clinical decisions [2, 23, 24], four guidelines [4, 25–27], one expert consensus [28], and four systematic reviews [12, 13, 29, 30]. The literature screening process is shown in Fig. 1. The general characteristics of the included literatures are shown in Table 2.

**Table 2:** General characteristics of the included literatures (*n* = 12)

Included literature	Year of publication (year)	Literature reference	The literature theme	Type of literature
Manaker et al. [2]	2024	UpToDate	Use of vasopressors and inotropes	Clinical decision
Paulsen et al. [23]	2024	UpToDate	Intravenous infusion devices for perioperative use	Clinical decision
Frank [24]	2023	UpToDate	Peripheral venous access in adults	Clinical decision
Nickel et al. [26]	2024	INS	Infusion Therapy Standards of Practice	Guideline
Evans et al. [4]	2021	PubMed	Surviving Sepsis Campaign: International Guidelines for Management of Sepsis and Septic Shock 2021	Guideline
Intensive Care Society. [25]	2020	ICS	Guidance for: The use of vasopressor agents by peripheral intravenous infusion in adult critical care patients	Guideline
WA Country Health Service. [27]	2018	WACHS	Peripheral Vasopressor Infusion Guideline - Adults	Guideline
Chinese Nursing Association. [28]	2021	Chinese Nursing Association	Nursing care of patients with infusion of vasoactive agents	Expert consensus
Owen et al. [29]	2021	PubMed	Adverse events associated With administration of vasopressor medications Through a peripheral intravenous catheter: a systematic review and meta-analysis	Systematic review
Tran et al. [12]	2020	PubMed	Complication of vasopressor infusion through peripheral venous catheter: A systematic review and meta-analysis	Systematic review
Tian et al. [13]	2020	PubMed	Safety of peripheral administration of vasopressor medications: A systematic review	Systematic review
Loubani et al. [30]	2015	PubMed	A systematic review of extravasation and local tissue injury from administration of vasopressors through peripheral intravenous catheters and central venous catheters	Systematic review

### Quality evaluation results of the included literature

For all the literature evaluations in this study, the ICC values of the investigators were > 0.750, indicating the high consistency and good reliability between the evaluators.

**Quality evaluation results of clinical decisions** Three clinical decisions [2, 23, 24] were included in this study. These articles met the evidence development criteria. Eight items were answered as ‘yes’, and the fourth evaluation item (‘Are the search methods transparent and comprehensive?’) and the fifth evaluation item(‘Is the evidence graded and is the system transparent and translatable?’) were‘No’.Therefore, all three clinical decisions were considered high quality and could be included. The results of the methodological quality assessment of the clinical decisions are shown in Table 3.

**Table 3:** Quality evaluation results of clinical decisions (*n* = 3)

Items	Manaker et al. [2]	Paulsen et al. [23]	Frank [24]
1. Is the summary specific in scope and application?	Yes	Yes	Yes
2. Is the authorship of the summary transparent?	Yes	Yes	Yes
3. Are the reviewer(s)/editor(s) of the summary transparent?	Yes	Yes	Yes
4. Are the search methods transparent and comprehensive?	No	No	No
5. Is the evidence graded and is the system transparent and translatable?	No	No	No
6. Are the recommendations clear?	Yes	Yes	Yes
7. Are the recommendations appropriately cited?	Yes	Yes	Yes
8. Are the recommendations current?	Yes	Yes	Yes
9. Is the summary free of possible bias?	Yes	Yes	Yes
10. Can this summary be applied to your patient(s)?	Yes	Yes	Yes

**Quality evaluation results of guidelines** Four guidelines were included in this study [4, 25–27]. The scores of the three guidelines in each field were ≥ 60%, and the recommendation level was A level [4, 25, 26] while the other guidelines were level B [27]. The results of the methodological quality assessment of the guidelines are shown in Table 4.

**Table 4:** Quality evaluation results of clinical guidelines (*n* = 4)

Guideline	Standardized scores in various domains (%)									
	Scope and purpose	Stakeholder involvement	Rigour of development	Clarity of presentation	Applicability	Editorial independence	≥ 60%	≥ 30%	ICC	Quality evaluation
Nickel et al. [26]	94.44%	75.00%	89.06%	95.83%	84.36%	66.67%	6	6	0.826	A
Evans et al. [4]	95.83%	79.17%	89.58%	91.67%	78.13%	77.08%	6	6	0.836	A
Intensive Care Society. [25]	87.50%	79.17%	72.40%	81.94%	77.08%	64.58%	6	6	0.864	A
WA Country Health Service. [27]	79.17%	69.44%	58.85%	79.17%	60.42%	50.00%	4	6	0.875	B

**Quality evaluation results of expert consensus** One expert consensus was included [28]. Table 5 contains the quality evaluation results of this expert consensus. This expert consensus had item 1 and item 5 as ‘No’, item 4 rated as ‘Unclear’, and the rest as ‘Yes’.Therefore, the expert consensus was considered high quality and could be included in the summary.

**Table 5:** Quality evaluation results of the expert consensus (*n* = 1)

Items	Chinese Nursing Association [28]
1. ls the source of the opinion clearly identified?	No
2. Does the source of opinion have standing in the field of expertise?	Yes
3. Are the interests of the relevant population the central focus of the opinion?	Yes
4. ls the stated position the result of an analytical process, and is there logic in the opinion expressed?	Unclear
5. ls there reference to the extant literature?	No
6. ls any in congruence with the literature/sources logically defended?	Yes

**Quality evaluation results of systematic reviews** Four systematic reviews [12, 13, 29, 30] were included, and Table 6 shows the quality evaluation results for these systematic reviews. All four systematic reviews were performed in the past 10 years. The four systematic reviews were all associated with the safe management of vasopressors through peripheral intravenous catheters. Owen et al. [29] Tran et al. [12] and Tian et al. [13] evaluated item 9 as ‘No’, and the rest as ‘Yes’; Loubani et al. [30] had item 5, item 6, and item 9 as ‘No’, and the rest as ‘Yes’; thus, all four documents were high quality and were included.

**Table 6:** Quality evaluation results of systematic reviews (*n* = 4)

Items	Owen et al. [29]	Tran et al. [12]	Tian et al. [13]	Loubani et al. [30]
1. Whether the evidence-based questions raised are clear and unambiguous?	Yes	Yes	Yes	Yes
2. Whether the literature inclusion criteria were appropriate for this evidence-based question?	Yes	Yes	Yes	Yes
3. ls the search strategy appropriate?	Yes	Yes	Yes	Yes
4. The adequacy of databases or resources for searching literature?	Yes	Yes	Yes	Yes
5. Whether the literatur equality evaluation criteria used are appropriate?	Yes	Yes	Yes	No
6. Whether two or more reviewers independently complete the quality evaluation of the literature?	Yes	Yes	Yes	No
7. Whether to take certain measures to reduce errors when extracting data?	Yes	Yes	Yes	Yes
8. Whether the methods of pooling studies were appropriate?	Yes	Yes	Yes	Yes
9. Whether the possibility of publication bias was assessed?	No	No	No	No
10. Whether the proposed policy or practice recommendations are based on the results of systematic reviews?	Yes	Yes	Yes	Yes
11. Whether the proposed directions for further research are appropriate?	Yes	Yes	Yes	Yes

### Summary and description of evidences

We summarized 29 pieces of best evidence, which included training and education, infusion site selection, vascular access placement, infusion regimen optimization, and complication management, as shown in Table 7. Because of the particularity of vasoconstrictors, most of the studies were observational studies, therefore only 31% (9/29) of the evidence was level 1.

In the training and education, there are two pieces of evidence, both first-class and strongly recommended. It is suggested to strengthen the standardized training for nursing staff and individualized education for patients.

There are six pieces of evidence in the infusion site selection, with five strong recommendations. It is suggested to establish vascular access in the forearm of the upper limb first, followed by the lower limb veins, and neck veins should only be considered in emergencies or when other sites are inaccessible, while avoiding the tube in special parts.

In the vascular access placement, nine pieces of evidence exist, including two pieces of first-class evidence, but one of them was weakly recommended. It is suggested to catheterization by experienced staff. Application of skin adhesive at puncture sites and catheter hubs is suggested for fixation, but adhesive-related skin injuries should result.

There are two pieces of first-class evidence among the eight pieces of evidence for infusion regimen optimization. Smart pumps with standardized drug libraries are recommended for precise delivery, and prompt response to alarms is advised. Administration of only one vasopressor per peripheral line is recommended.

In complication management, four pieces of evidence exist, with three pieces of first-class evidence. It is suggested to prompt management of complications such as extravasation and phlebitis.

**Table 7:** Summary of best evidence for safe management of vasopressors through peripheral intravenous catheters

Category	Content of evidence	Level	Recommendation level
Training and Education	1. It is recommended to develop standardized protocols for safe peripheral vasopressor infusion and enhance nurse training [12, 25–27].	1	A
	2. Education plans tailored to patients and caregivers are recommended, addressing age, cognitive level, health literacy, and access to educational resources to emphasize infusion precautions [26, 28].	1	A
Infusion site selection	3. Veins > 4 mm in diameter are recommended for catheterization [12, 26].	3	B
	4. The forearm veins (e.g., cephalic, basilic, and median cubital) in the upper limb are strongly recommended as the primary access sites [4, 12, 13, 24, 26, 27, 30].	3	A
	5. Lower limb veins (e.g., great saphenous or dorsal metatarsal veins) are recommended if upper limb access is unavailable [24].	5	A
	6. Neck veins should only be considered in emergencies or when other sites are inaccessible [24, 26, 30].	3	A
	7. It is recommended to avoid joints, infected/burned/edematous areas, arteriovenous fistulas, blood pressure cuff sides, or planned surgical sites [24, 26, 27].	5	A
	8. Temporary catheterization of limbs with motor/sensory impairments is conditionally recommended under close monitoring [24].	5	A
Vascular access placement	9. Catheterization by experienced staff is recommended to improve first-attempt success rates [24, 26].	1	A
	10. The use of ultrasound or near-infrared guidance is recommended for difficult vascular access [4, 12, 24–26].	3	A
	11. Catheters ≥ 20G are recommended, with ≥ 2/3 of the catheter length placed intravascularly [12, 24–26, 29].	3	A
	12. Catheters with a length of 2–3 cm are routinely selected, and long catheters with a length of 5–15 cm are recommended for cubital fossa treatment [24].	4	A
	13. Splinting is recommended to stabilize catheters placed near joints [24, 26].	5	B
	14. Application of skin adhesive at puncture sites and catheter hubs is suggested for fixation [24].	1	B
	15. Transparent dressings are recommended for catheter securement [24, 26].	3	A
	16. Blood return confirmation and flushing with 5–10 mL 0.9% sodium chloride post-placement are recommended [12–13, 25, 27].	3	A
	17. Repeated punctures at the same site should be avoided [23, 24–27, 30].	4	A
Infusion regimen optimization	18. Continuous peripheral vasopressor infusion is recommended to be limited to ≤ 24 h, with extensions permitted only under close monitoring [2, 4, 12, 13, 26, 29, 30].	3	B
	19. It is recommended to use the lowest effective vasopressor concentrations and doses [25, 26].	3	A
	20. The following concentrations are suggested: norepinephrine 16 µg/mL, epinephrine 16 µg/mL, phenylephrine 100 µg/mL, metaraminol 0.5 mg/mL [25].	3	B
	21. The following maximum dose are suggested: norepinephrine 25 µg/min, epinephrine 25 µg/min, phenylephrine 10.8 mg/h, metaraminol 10 mg/h [12, 25].	3	B
	22. Vascular patency verification before infusion is recommended [24, 26, 27].	5	A
	23. Smart pumps with standardized drug libraries are recommended for precise delivery, and prompt response to alarms is advised [23, 25–28].	1	A
	24. Administration of only one vasopressor per peripheral line is recommended [26, 28].	1	A
	25. Hourly assessment and documentation of catheter function are recommended [12, 26, 27].	3	A
Complication management	26. For extravasation, immediate cessation of infusion, aspiration of residual drug, catheter removal, and marking of the affected area are recommended [12, 13, 23–27].	1	A
	27. Subcutaneous phentolamine injection and limb elevation are recommended for extravasation management; terbutaline or topical nitroglycerin are suggested alternatives if phentolamine is unavailable [2, 12, 13, 24–26, 29].	1	A
	28. For phlebitis, immediate infusion cessation, cold or heat therapy, limb elevation, and analgesics or anti-inflammatories as needed are recommended [24–26].	1	A
	29. Documentation and analysis of infusion-related adverse events are recommended [25–27].	3	A

## Discussion

Traditionally, the CVC has been the preferred route for vasopressor administration. However, its use in emergencies is limited by the technical skill required for insertion, the risk of potentially severe complications, and significant treatment delays during the procedure. In contrast, PIVC insertion is simpler and faster, significantly reducing medication start time. This enables rapid hemodynamic stabilization, shortens the duration of tissue hypoperfusion, and is associated with a significantly lower 28-day mortality risk (18.3% vs. 38.7%) [31]. Recent high-quality evidence and guidelines [2, 24–27] clearly state that vasopressor infusion through PIVC is a safe alternative when strict protocols are followed. It is particularly suitable during the initial resuscitation of hemodynamically unstable patients and should be prioritized over delaying treatment while awaiting CVC placement.

This study systematically integrated 29 evidence-based recommendations into a structured, comprehensive bundle of interventions. These cover critical aspects of safe PIVC vasopressor administration. Compared to fragmented, single-intervention suggestions in prior research, this integrated framework more effectively reduces complication risks like extravasation and phlebitis caused by improper technique. This ensures early hemodynamic stability while minimizing iatrogenic injury [32, 33], effectively preventing related vascular and tissue damage. Ultimately, it helps reduce patient complication rates and mortality.

Despite these well-established benefits, current research indicates inconsistencies between clinical practices and guidelines for peripheral vasopressor infusion, potentially leading to complications such as phlebitis and extravasation. This highlights the need for evidence-based approaches to enhance nursing practices and bridge theory-practice gaps.

### Standardized training and education ensure safe infusion

Evidence 1–2 emphasizes the importance of standardized nurse training and patient-specific education. Vasopressors, with their potent vasoconstrictive effects, can cause complications like extravasation, phlebitis, or tissue necrosis if improperly administered, directly impacting patient safety and outcomes [34]. Nurses’ expertise and operational skills critically determine infusion safety [15]. Thus, establishing standardized protocols for peripheral vasopressor infusion and enhancing nurse training are essential [12, 25–27]. Training should cover pharmacological properties of vasopressors, infusion device usage, vascular access establishment and maintenance, early management of complications, etc [25–29]. Training formats should combine lectures, simulations, and clinical practice, reinforced by regular assessments and feedback [35]. Virtual reality simulations could improve nurses’ ability to handle complex scenarios [36].

Patient and caregiver education is equally vital. The study [37] showed that anxiety, low health literacy, and poor compliance increase the risks of complications like extravasation. Tailored education programs should account for age, cognitive level, health literacy, and access to educational resources [26, 28]. Visual aids, family involvement, and bedside demonstrations may enhance understanding for elderly or less-educated patients [38–40]. Education should focus on treatment goals, infusion precautions, and emergency responses [26]. The synergistic effects of standardized training and individualized education can enhance infusion safety. Future exploration of AI-guided real-time systems could further optimize training and education strategies.

Healthcare institutions can implement appropriate training and education measures based on their resources. Teaching hospitals or large medical centers may establish comprehensive simulation training centers utilizing VR technology for high-risk scenario drills, develop online learning platforms and knowledge repositories, and appoint dedicated nurse educators for patient education. Primary care hospitals or resource-limited settings can utilize standardized video training packages for foundational nurse training, employ simple illustrated pamphlets for patient education, and form regional collaborative networks to share training resources.

### Optimal infusion site selection forms the foundation of safety

Evidence 3–8 outlines strategies for infusion site selection. Vein diameter, blood flow rate, and anatomical features influence infusion safety. Evidence 3 recommends selecting veins with a diameter > 4 mm because faster blood flow dilutes the drug and reduces vascular irritation [12, 26]. However, the strength of this recommendation is ‘weak’ due to limited generalizability. Considerable variation exists in vein diameter across individuals, anatomical locations, and physiological states. Accurate assessment often requires ultrasound guidance, but not all operators possess the necessary expertise or access to equipment [41]. The forearm is recommended as the preferred infusion site due to its limited mobility, which reduces catheter displacement caused by limb movement. The superficial and stable positioning of upper limb veins, combined with their faster blood flow, minimizes local irritation [42]. Suitable veins in this region include the cephalic, basilic, and median veins [4, 12, 13, 26]. When upper limb access is unfeasible, the great saphenous vein or dorsal metatarsal veins of the lower limbs may serve as alternatives [24]. However, slower blood flow in these vessels increases the risk of drug accumulation. Notably, prolonged immobilization after lower limb catheterization should be avoided to prevent deep vein thrombosis [43].

Special site selection must strictly follow clinical indications. Neck vein catheterization is technically challenging and adjacent to critical neurovascular structures, making it suitable only for emergencies or when other sites are inaccessible [24, 26, 30]. Additionally, avoid establishing vascular access in joints, infected/burned/edematous areas, arteriovenous fistulas, blood pressure cuff sides, or planned surgical sites [24, 26, 27]. Frequent joint movement increases catheter displacement risks. Infected or burned areas may exacerbate local inflammation. Edematous tissues hinder successful puncture and observation due to high tissue tension. Arteriovenous fistulas exhibit abnormal vascular structures, while blood pressure cuff placement compromises local circulation, both impeding drug delivery. Catheterization at planned surgical sites may interfere with procedures and raise infection risks. For limbs with motor or sensory impairments, temporary catheterization under close monitoring is permissible, but alternative sites should be promptly evaluated and prioritized to mitigate long-term risks [24]. In clinical practice, infusion site selection requires dynamic adjustments based on individual patient characteristics.

In clinical practice, infusion site selection requires ongoing adjustment based on individual patient characteristics. Clinical departments should develop and display clear site selection flowcharts in treatment areas. They should also enforce training in visual inspection and palpation skills for vein assessment. Finally, competency in ultrasound-guided catheterization should be incorporated into core skills training.

### Proper vascular access placement ensures safe infusion

Evidence 9–17 summarizes key technical considerations for vascular access safety. First-attempt success is critical to minimize vascular injury, and catheterization should be performed by experienced staff [24, 26]. For patients with difficult vascular access (e.g., dehydration, obesity, or edema), ultrasound or near-infrared technology is recommended [4, 12, 24–26]. These methods provide real-time visualization of vessel location, course, and depth, enabling precise placement and reducing repeated puncture-related trauma [44]. Catheter size and length must match vascular conditions. Catheters ≥ 20G are preferred to reduce infusion resistance [12, 24–26, 29]. Ensure ≥ 2/3 of the catheter remains intravascular to minimize local irritation and displacement risks [25, 26]. At cubital sites, longer catheters (5–15 cm) are advised to reduce displacement caused by joint movement and uneven vascular pressure [24].

Proper fixation and maintenance are equally critical. Joints have greater mobility, which increases the risk of catheter displacement or dislodgement. Using splints to secure catheters in joint areas can effectively restrict movement, but may reduce flexibility in adjusting limb angles during puncture [24, 26]. Applying skin adhesive at both the puncture site and catheter hub enhances fixation [24], but adhesive-related skin injuries should be monitored [45]. Evidence 13 recommends splinting catheters in joint areas to restrict movement. Although splinting limits mobility, it may increase manipulation complexity during catheter repositioning, raise the risk of skin pressure injuries, and often cause overlooked patient discomfort. Therefore, this recommendation received a ‘weak’ strength rating [24, 26]. Evidence 14 suggests applying skin adhesive agents at the puncture site and catheter hub to enhance securement [24]. However, PIVC dwell times are typically short, and high-quality transparent dressings usually provide sufficient adhesion. Additional adhesive use significantly increases the risk of medical adhesive-related skin injury upon removal [45] and adds cost. Consequently, this evidence also received a ‘weak’ strength rating. Use adhesive agents cautiously only when both of the following conditions are present: inadequate dressing adhesion (such as with excessive sweating or oily skin) and a critically important catheter position. When adhesives are necessary, select low-allergenic formulations and apply adhesive removers during dressing changes. Blood return upon catheter insertion confirms proper placement, followed by easy flushing with 5–10 mL 0.9% sodium chloride to ensure patency [12, 13, 25, 27]. Transparent dressings are recommended post-confirmation for continuous site observation [24, 26]. Repeated punctures at the same site increase vascular damage, local inflammation, and phlebitis risks [46]. Avoid re-puncturing areas with hematomas from prior failed attempts [24, 25, 27, 30].

In practice, safe vascular access relies on skilled catheterization, imaging-assisted techniques, appropriate device selection, and secure fixation. Combined, these measures reduce complications and ensure safe peripheral vasopressor infusion. Clinical departments should implement ongoing quality monitoring of first-attempt insertion success rates and puncture attempt frequencies. Concurrently, they need to develop standardized criteria for identifying difficult vascular access cases with clear pathways for specialist consultation. Additionally, ensuring reliable access to adequate inventories of high-quality catheters and dressings is essential for maintaining consistent clinical practice standards.

### Scientific infusion protocols are critical for safety

Evidence 18–25 provides recommendations for infusion protocols, including duration, drug concentration/dose, device use, and monitoring. Current evidence lacks consensus on safe infusion durations for peripheral vasopressors. Prolonged infusion causes continuous vascular endothelial stimulation, leading to dysfunction and structural damage, increasing risks of phlebitis and extravasation [47]. Evidence 18 suggests limiting continuous peripheral vasopressor infusion to ≤ 24 hours. Extensions require close monitoring of vascular tolerance [2, 12, 29, 30]. However, this recommendation’s operational parameters remain ambiguous due to undefined monitoring frequencies, and unspecified duration thresholds for extensions. Therefore, this recommendation received a ‘weak’ strength rating. To enhance clinical utility, healthcare institutions should establish explicit monitoring protocols, while implementing formal review processes mandating documented verification of vascular tolerance and hemodynamic stability for infusions exceeding 24 h.

Peripheral veins have slower flow rates than central veins, necessitating lower drug concentrations and doses to achieve therapeutic efficacy while minimizing local vascular irritation [25, 26]. No standardized thresholds exist for safe vasopressor concentrations or doses. Limited studies explore norepinephrine, epinephrine, phenylephrine, and metaraminol safety profiles, but evidence quality remains low, warranting further research [12, 25]. Clinically, adjust concentrations and doses dynamically based on skin condition and blood pressure fluctuations to balance efficacy and safety. Limited studies have examined the maximum safe concentrations and doses for norepinephrine, epinephrine, phenylephrine, and metaraminol, but existing evidence derives solely from observational research. Multicenter randomized controlled trials are needed to establish therapeutic windows for specific vasopressors through PIVC across diverse patient populations and to develop personalized predictive models [12, 25]. Furthermore, Evidence 20–21 received ‘weak’ recommendation grades because their fixed numerical thresholds fail to account for interpatient variability in drug metabolism and response. Drug concentration selection requires continuous balancing between local venous integrity and hemodynamic stability, necessitating individualized titration. While these recommendations provide initial guidance, clinicians must dynamically adjust concentrations and doses based on individual patient characteristics, continuous vital sign monitoring, and pharmacological response.

Ensure vascular patency before each infusion to prevent complications like extravasation [24, 26, 27]. Smart pumps with standardized drug libraries improve safety through precise delivery, alarms, and data logging [23, 25–28]. Respond immediately to “high pressure” or “occlusion” alarms. However, subcutaneous tissue compliance may mask subtle pressure changes from extravasation. Therefore, infusion pump alarms cannot be solely relied upon, hourly catheter function assessment remains essential for early complication detection [12, 23, 26, 27]. Each peripheral line should deliver only one vasopressor to avoid drug interactions and excessive local vasoconstriction, which heighten vascular injury and extravasation risks [26, 28].

Clinical practice should incorporate smart infusion pumps with enabled drug libraries. Include smart pump alarm response times in quality control metrics and conduct regular drug library reviews. Establish a clear ‘one-drug-one-tube’ policy prohibiting multi-drug infusion through single lumens. Implement standardized checklists and documentation protocols for hourly catheter assessments. Emphasize the critical importance of dynamic assessment and adjustment throughout infusion therapy.

### Timely complication management is essential

Evidence 26–29 details the management of complications such as extravasation and phlebitis. Extravasation, the most frequent complication of peripheral vasopressor infusion, initially presents as pain, swelling, and erythema at the puncture site, progressing to tense blisters, tissue hardening, or necrosis [48]. Immediate actions upon extravasation include stopping the infusion, aspirating residual drug, removing the catheter, and marking the affected area. Phentolamine, an α-receptor antagonist, reverses vasoconstriction when administered locally within 12 h, reducing ischemic risks. If unavailable or ineffective, terbutaline (smooth muscle relaxant) or topical nitroglycerin (PI3K/Akt pathway activator) may be alternatives to promote vasodilation [2, 12, 13, 23–26, 29]. Necrotic tissue requires debridement, antibiotic therapy, and wound rehabilitation [49]. Chemical phlebitis, characterized by redness, pain, or cord-like induration, may progress to necrosis [50]. Management includes immediate infusion cessation. Apply cold compresses within 24 h to reduce acute inflammation, then switch to warm compresses to promote fluid dispersion and reabsorption. Analgesics and anti-inflammatories should be administered as needed [24–26]. Document and report infusion-related adverse events. Conduct failure mode analysis and apply PDCA cycles to refine protocols and reduce recurrence [25–27].

Clinical practice should develop and prominently display clear emergency response flowcharts for managing extravasation or phlebitis. Essential antidote kits containing agents like phentolamine must be readily available in treatment areas. Institutions need to establish standardized systems for reporting and analyzing such events. Event reporting rates and response timeliness should be incorporated into quality control metrics. Finally, analyzed findings should be systematically shared to promote institution-wide learning.

### Limitations

Although this study comprehensively summarizes evidence on peripheral vasopressor infusion safety, findings may be influenced by racial, regional, and cultural differences. Additionally, only English and Chinese publications were included, and the exclusion of studies in other languages may introduce potential language bias. During evidence implementation, institutions should tailor recommendations by integrating patient-specific factors and clinical contexts to develop optimal safety protocols. Future research should monitor emerging evidence and refine guidelines to enhance clinical relevance.

## Conclusion

This study systematically synthesized 29 evidence-based recommendations for safe peripheral vasopressor administration. These aim to rapidly restore and maintain hemodynamic stability while minimizing iatrogenic vascular and tissue injury through precise PIVC management. Given the multinational origin and implementation complexity of this evidence, healthcare institutions must critically evaluate its feasibility, appropriateness, clinical relevance, and effectiveness within local contexts before adoption, developing tailored implementation protocols. Future research should prioritize addressing critical evidence gaps, particularly establishing individualized safe drug concentrations, dosage thresholds, and infusion durations, while optimizing technical applications to continually refine clinical guidelines.

### Relevance to clinical practice

Safe peripheral vasopressor infusion remains a significant clinical challenge. This study synthesizes 29 evidence-based recommendations addressing five critical domains: training and education, infusion site selection, vascular access placement, infusion regimen optimization, and complication management. These interconnected components form a comprehensive safety framework. Standardized training ensures clinician competency, appropriate site selection establishes a stable foundation for infusion, proper vascular access placement enables safe drug delivery, optimized protocols minimize risks during administration, and prompt complication management mitigates adverse outcomes. Together, these measures create a systematic approach to peripheral vasopressor administration. Implementing these recommendations can guide healthcare providers globally in enhancing patient safety, reducing complications, and improving clinical outcomes during peripheral vasopressor therapy.

## Electronic supplementary material

Below is the link to the electronic supplementary material.

Supplementary Material 1

## Data Availability

No datasets were generated or analysed during the current study.

## Abbreviations

AGREE II: Appraisal of Guidelines for Research and Evaluation
CASE: Critical Appraisal for Summaries of Evidence
CVC: Central venous catheters
GIN: Guidelines International Network
ICC: Intraclass correlation coefficient
INS: Intravenous Nurses Society
JBI: Joanna Briggs Institute
NICE: National Institute for Health and Clinical Excellence
PIVCs: Peripheral intravenous catheters
RNAO: Registered Nurses’ Association of Ontario
SIGN: Scottish Intercollegiate Guidelines Network
