# Supplementary material for: Summary of best evidence for safe management of vasopressors through peripheral intravenous catheters
Source: BMC Nurs. 2025 Jul 31;24:1000. doi: 10.1186/s12912-025-03635-3 (PMC12312446; doi:10.1186/s12912-025-03635-3)
Supplement: Supplementary file 1 — Supplementary Material 1 [file 12912_2025_3635_MOESM1_ESM.docx]

**APPENDICES**

**Appendix 1.PubMed search strategy**

(vasoconstrictor agents[MeSH Terms]) OR (vasoconstrictor*[Title/Abstract] OR vasopressor*[Title/Abstract] OR vasoactive agonist*[Title/Abstract] OR vasoactive agent*[Title/Abstract] OR epinephrine[Title/Abstract] OR norepinephrine[Title/Abstract] OR dopamine[Title/Abstract] OR dobutamine[Title/Abstract])

AND

(peripheral catheterization[MeSH Terms]) OR (peripheral catheterization*[Title/Abstract] OR peripheral venous catheter*[Title/Abstract] OR peripheral intravenous[Title/Abstract] OR indwelling needle[Title/Abstract])

**Appendix 2.Embase search strategy**

(vasoconstrictor*:ti,ab,kw OR vasopressor*:ti,ab,kw OR 'vasoactive agonist*':ti,ab,kw OR 'vasoactive agent*':ti,ab,kw OR epinephrine:ti,ab,kw OR norepinephrine:ti,ab,kw OR dopamine:ti,ab,kw OR dobutamine:ti,ab,kw)

AND

(peripheral catheterization*:ti,ab,kw OR peripheral venous catheter*:ti,ab,kw OR peripheral intravenous:ti,ab,kw OR indwelling needle:ti,ab,kw)

**Appendix 3. Web of science search strategy**

(TS=(vasoconstrictor agents) OR TI=(vasoconstrictor* OR vasopressor* OR vasoactive agonist* OR vasoactive agent* OR epinephrine OR norepinephrine OR dopamine OR dobutamine))

AND

(TS=(peripheral catheterization) OR AB=(peripheral catheterization* OR peripheral venous catheter* OR peripheral intravenous OR indwelling needle))

**Appendix 4. CINAHL (EBSCOhost) search strategy**

SU(vasoconstrictor agents) OR XB(vasoconstrictor* OR vasopressor* OR vasoactive agonist* OR vasoactive agent* OR epinephrine OR norepinephrine OR dopamine OR dobutamine)

AND

SU(peripheral catheterization) OR XB(peripheral catheterization* OR peripheral venous catheter* OR peripheral intravenous OR indwelling needle))

**Appendix 5.Cochrane Library search strategy**

(vasoconstrictor agents:MeSH descroptor) OR (vasoconstrictor*:ti,ab,kw OR vasopressor*:ti,ab,kw OR 'vasoactive agonist*':ti,ab,kw OR 'vasoactive agent*':ti,ab,kw OR epinephrine:ti,ab,kw OR norepinephrine:ti,ab,kw OR dopamine:ti,ab,kw OR dobutamine:ti,ab,kw)

AND

(peripheral catheterization:MeSH descroptor) OR (peripheral catheterization*:ti,ab,kw OR peripheral venous catheter*:ti,ab,kw OR peripheral intravenous:ti,ab,kw OR indwelling needle:ti,ab,kw)

**Appendix 6.BMJ, UpToDate, DynaMed, JBI, GIN, NICE, SIGN,RNAO, and INS search strategy**

vasoconstrictor agent AND peripheral catheterization

vasoconstrictor AND peripheral catheterization

vasopressor AND peripheral catheterization

vasoactive agonist AND peripheral catheterization

vasoactive agent AND peripheral catheterization

epinephrine AND peripheral catheterization

norepinephrine AND peripheral catheterization

dopamine AND peripheral catheterization

dobutamine AND peripheral catheterization

vasoconstrictor agent AND peripheral venous catheter

vasoconstrictor AND peripheral venous catheter

vasopressor AND peripheral venous catheter

vasoactive agonist AND peripheral venous catheter

vasoactive agent AND peripheral venous catheter

epinephrine AND peripheral venous catheter

norepinephrine AND peripheral venous catheter

dopamine AND peripheral venous catheter

dobutamine AND peripheral venous catheter

vasoconstrictor agent AND peripheral intravenous

vasoconstrictor AND peripheral intravenous

vasopressor AND peripheral intravenous

vasoactive agonist AND peripheral intravenous

vasoactive agent AND peripheral intravenous

epinephrine AND peripheral intravenous

norepinephrine AND peripheral intravenous

dopamine AND peripheral intravenous

dobutamine AND peripheral intravenous

**Appendix 7.Chinese Medical Journal Full-text Database, Sinomed, CNKI, Wanfang, and VIP search strategy**

Title/Abstract (缩血管药 OR 血管加压药 OR 血管活性药 OR 肾上腺素 OR 去甲肾上腺素 OR 多巴胺 OR 多巴酚丁胺)

AND

Title/Abstract(外周静脉 OR 外周导管 OR 外周置管 OR 留置针)

**Appendix 8.Chinese Nursing Association search strategy**

血管活性药 AND 外周静脉

血管活性药 AND 外周导管

缩血管药 AND 外周静脉

缩血管药 AND 外周导管

血管加压药 AND 外周静脉

血管加压药 AND 外周导管
